# From Mouth-to-Mouth to Bag-Valve-Mask Ventilation: Evolution and Characteristics of Actual Devices—A Review of the Literature

**DOI:** 10.1155/2014/762053

**Published:** 2014-05-27

**Authors:** Abdo Khoury, Sylvère Hugonnot, Johan Cossus, Alban De Luca, Thibaut Desmettre, Fatimata Seydou Sall, Gilles Capellier

**Affiliations:** ^1^Department of Emergency Medicine & Critical Care, University of Franche-Comté, Medical Center, 25000 Besançon, France; ^2^Inserm CIC-1431, University of Franche-Comté, Medical Center, 25000 Besançon, France; ^3^Monash University, Melbourne, VIC 3800, Australia

## Abstract

Manual ventilation is a vital procedure, which remains difficult to achieve for patients who require ventilatory support. It has to be performed by experienced healthcare providers that are regularly trained for the use of bag-valve-mask (BVM) in emergency situations. We will give in this paper, a historical view on manual ventilation's evolution throughout the last decades and describe the technical characteristics, advantages, and hazards of the main devices currently found in the market. Artificial ventilation has developed progressively and research is still going on to improve the actual devices used. Throughout the past years, a brand-new generation of ventilators was developed, but little was done for manual ventilation. Many adverse outcomes due to faulty valve or misassembly were reported in the literature, as well as some difficulties to ensure efficient insufflation according to usual respiratory parameters. These serious incidents underline the importance of BVM system routine check and especially the unidirectional valve reassembly after sterilization, by only experienced and trained personnel. Single use built-in devices may prevent disassembly problems and are safer than the reusable ones. Through new devices and technical improvements, the safety of BVM might be increased.

## 1. Introduction

Ventilation, used to deliver supplemental oxygenation to respiratory-failing patients, is a crucial procedure. It was described ages ago, and since that time, techniques and devices used are continuously improving. Claudius Galenus was among the first to talk about lungs and ventilation almost 2000 years ago [1] and since then, several scientists and philosophers have tried to understand this concept [2]. At the middle of the 20th century, several unidirectional valves were developed with different characteristics. However, many different manual ventilation methods were described and used including mouth-to-mouth and mouth-to-nose but the bag-valve-mask (BVM) technique remains the commonly used one in emergency situations and in prehospital settings [3]. This paper draws a historical view of manual ventilation's evolution throughout the last decades and describes the technical characteristics, advantages, and hazards of the main systems currently used for manual ventilation.

## 2. History of Artificial Ventilation

Ventilation with BVM is the commonly used technique to provide manual positive pressure ventilation to respiratory-failing patients. From the mid-1500s until the early 1900s, artificial ventilation techniques reported in the literature recall only mouth-to-mouth and the use of bellows [1]. Indeed, in 1472, Paulus Bagellardus published the first known book on childhood diseases and described mouth-to-mouth resuscitation by recommending to midwives to blow into the newborn's mouth if there is no respiration [2, 4]. This shows that mouth-to-mouth ventilation was already considered at that time. In 1543, after further investigations on a porcine model, Andreas Vesalius advised to provide air into the trachea with a reed for increasing animal's survival. This practice was taken over in 1559 by an Italian professor of anatomy Matteo Realdo Colombo who also described the tracheotomy's method. One century later, Robert Hooke, one of the greatest experimental scientists of the seventeenth century, repeated the Vesalius's experimentation using a strangled chicken model, which was ventilated by bellows. He demonstrated with this model that it was only the fresh air leak which caused death [1]. In 1732, the first mouth-to-mouth ventilation case was reported on a coal miner. This latter resuscitation was performed by the surgeon William Fossach [5]. He presented in 1744 at Edinburgh the case study of his mouth-to-mouth rescue [6]. In 1787, Baron Antoine Portal proposed, for respiratory insufficiency cases, to inflate the lungs of the new-born with air. The Scottish surgeon John Hunter, advocate of the experimental method in Medicine, who developed human bellows with pressure relief valve, recommended to the Royal Human Society in 1776 the need to apply artificial ventilation immediately for resuscitation [2, 6]. Furthermore, in order to reduce stomach inflation, the major problem with bellows ventilation, he suggested pressing gently the larynx against the vertebrae [2, 7]. The bellows ventilation was condemned by the Royal Human Society and the French Academy of Medicine for lack of safety due to their first adverse effects. In 1745, John Fothergill listed singular advantages of mouth-to-mouth expired air ventilation compared to the bellows ventilation during resuscitation [2, 6]. He said that “the warmth and moisture of the breath would be more likely to promote the circulation than the chilling air forced out of a pair of bellows and that the lungs of one man may bear, without injury, as great a force as those of another can exert, which by the bellows cannot always be determined” [2]. Indeed, with mouth-to-mouth ventilation, it is impossible to increase pressure to be higher than that the human is able to generate. Nevertheless, an example of successful bellows ventilation has been reported by Fell in 1891 in a clinical trial [1]. James Leroy d'Etiolles emphasized the need for early use of the bellows and recommended in 1828 a graduated bellows according to the patient size to reduce hyperventilation with high volumes which may induce barotrauma [1]. In 1958, Peter Josef Safar, “the father of modern resuscitation,” demonstrated the superiority of mouth-to-mouth ventilation over other methods of manual ventilation in a clinical study [8, 9].

At the middle of the 20th century, several unidirectional valves were developed with different technical characteristics. The original bag-valve-mask concept was developed in 1953 by the German doctor Holger Hesse and his partner Danish anesthetist Henning Ruben, following their initial work on a suction pump. Their resuscitator, named “Ambu” (Artificial Manual Breathing Unit), was manufactured and marketed in 1956 by their company [10].

## 3. Bag-Valve-Mask System

**Figure 1:**
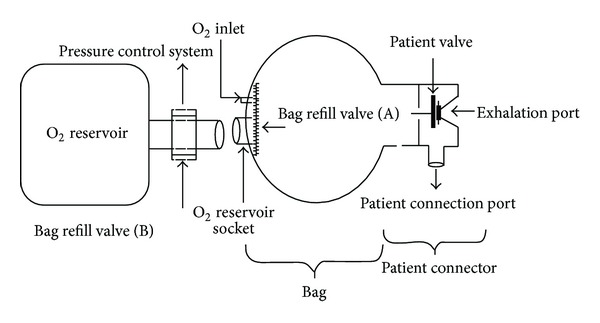
Basic components of the BVM system (De Godoy et al. [11]).

An air chamber (or bag) and a patient connector constitute the BVM system. The patient connector consists of a patient unidirectional valve, an expiratory port and a patient connection port. This latter is plugged to an interface, which can be either a mask or an endotracheal tube. An air volume is provided to the patient when the rescuer squeezes the bag. These different parts are depicted in Figure 1 [11].

## 4. Patient Valve Technical Characteristics

Unidirectional or one-way patient valves are nonrebreathing valves (NRVs), which have to be combined with self-inflating bags to be used as complete resuscitation devices. These valves are composed of an inspiratory and an expiratory port and permit either spontaneous or controlled respiration. Patient valves are used for positive pressure ventilation with a BVM or a mechanical ventilator [12]. In most cases, in order to minimize dead space, the valves are situated close to the patient's airway [13, 14]. Several NRVs are developed with different technical characteristics. Among them, we will describe succinctly Ambu and Laerdal valves, the most popular NRVs used.

### 4.1. Ambu Valves

**Figure 2:**
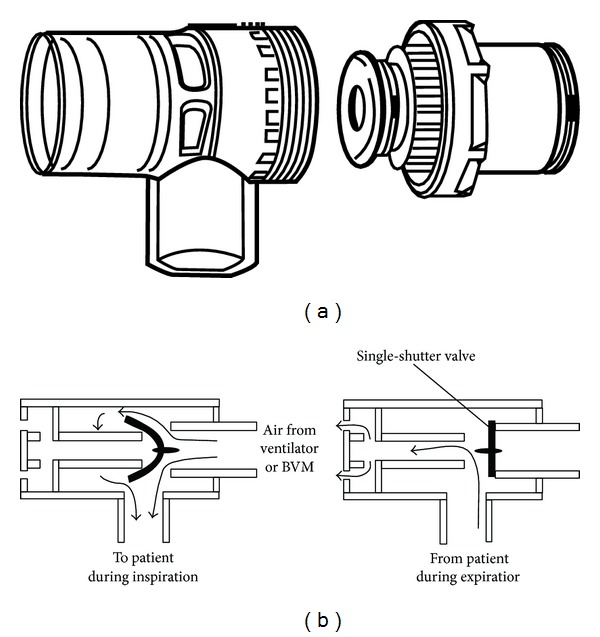
Ambu single-shutter valve ((a) http://helid.digicollection.org/, (b) Kim et al., 2008 [16]).

Ambu or Artificial Manual Breathing Unit valves are made of two unidirectional silicone rubber flaps (mushroom valves) constituted by an inspiratory and an expiratory flap. Usually, they are used with a flexible ventilation bag in the operating room. It is the oldest developed valve for ventilation. It presents a small dead space and low resistance to flow [15]. Many different Ambu valves are now available. An example of Ambu single-shutter valve type Ambu Mark III is depicted below (Figure 2).

### 4.2. Laerdal Valves

**Figure 3:**
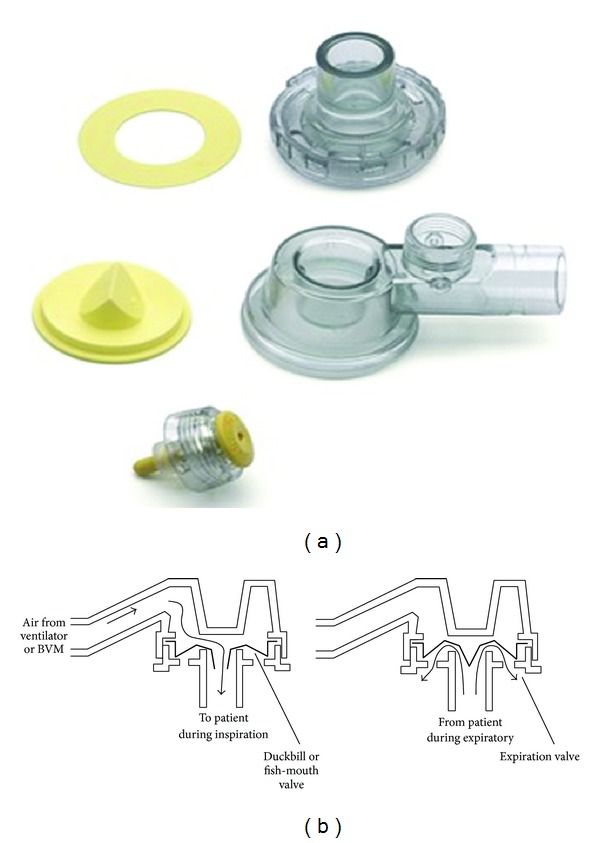
Laerdal valve ((a) http://www.laerdal.com/, (b): Kim et al., 2008 [16]).

These valves are used with self-inflating bags and have a particular “duckbill” shape or lip membrane constituted by a thin and flexible diaphragm and a flat silicone ring (Figure 3). The “duckbill” valve (inspiratory valve) opens during inspiration and also impinges upon a flat silicone ring (expiratory valve) that moves to close the exit port [13]. These valve types are the most commercially popular NRVs due to their easy incorporation into a wide variety of devices and remain the first choice in a large number of applications [17].

The different technical characteristics of these valves are presented in Table 1.

## 5. Bag-Valve-Mask System Drawbacks and Hazards

### 5.1. Nonrebreathing Valve Design

BVM systems with nonrebreathing valves can be used either in controlled ventilation or in spontaneous ventilation to keep or to increase patient arterial oxygen blood pressure prior to intubation [11, 18]. However, according to Tibballs et al., some devices with a “duckbill” valve should not be used to provide oxygen during spontaneous ventilation. These NRVs provide only a negligible oxygen flow when the patient's efforts fail to open the valve during inspiratory effort. Therefore, they recommended not using BVM with NRV along with mask or endotracheal tube (ETT) to provide oxygen during spontaneous ventilation except if the “duckbill” valve opening can be assured. Otherwise, the patient will inhale essentially expired gas [18]. Recently, Payne et al. simulated Laerdal and Ambu valve resistances over a range of constant flow conditions. For flows ranging from 5 to 45 L/min, the resistance of these valves induces a loss of pressure of less than 2.04 cm H_2_O which is still high compared to the limit fixed by the European Committee for Standardization (CEN) (1.53 cm H_2_O at a gas flow of 35 L/min) [14]. Furthermore, the best BVM system to deliver oxygen to spontaneously ventilating patients must have a low resistance valve and, in addition, an incorporated disc to prevent air entrainment [19]. However, “duckbill” valves did not reliably prevent air entrainment [19]. It remains, therefore, important to know the BVM characteristics before the use on a patient breathing spontaneously [11].

### 5.2. Nonrebreathing Valve Limits

BVM ventilation is quite difficult to perform and NRVs must be mounted correctly to provide adequate ventilation to the patient. Critical incidents have been documented and a large variety of causes have been identified. Several studies showed some accidents due to faulty one-way valves in BVM and mechanical ventilation [12, 20]. A recent study described a pulmonary barotrauma case due to the “locking” of the Ambu valve in the inspiratory position [20]. Another case study reported a faulty inspiratory diaphragm of the Laerdal NRV connected to a Dräger Oxylog ventilator which induced 79% SaO_2_ (down from 97%) in a patient. Indeed, they revealed that the “duckbill” valve was not moving totally into the inspiratory position at lower inspiratory flow rates and this caused major leaks and, thereby, much of the insufflated volume bypassed the patient and led to desaturation [12].

### 5.3. Bag-Valve-Mask System Misassembly

Many BVM misassembly problems have also been reported in the literature inducing inadequate tidal volumes, barotrauma, and potentially dangerous issues [12, 16, 21, 22]. Ho et al. described two exhalation obstruction cases due to the Laerdal valve misassembly, when two “fish-mouth” lips or “duckbill” valves were inserted instead of one [22]. In 2002, Smith reported a complete failure of an adult manual resuscitator and the inability to ventilate a cardiac arrest victim [21]. This was due to the “duckbill” valve missing in the patient's valve assembly [21]. Munford and Wishaw described, after an inadequate ventilation during a resuscitation attempt, another case of misassembly with an NRV used mainly in anesthesia (Ruben valve) [12]. Indeed, the Ambu bag was connected to the patient's port of a Ruben valve and not to the bag inlet, and thus each delivered insufflation passed out of the expiratory port [12]. Similar accidental valve inversions, with either a respiratory filter inadvertently inserted into the expiratory port or an insertion of the bag reservoir into the patient port, were encountered with Ambu A valves [23]. In order to prevent these serious problems of connection, international standards and French regulations, published in 1996, prohibit the use and marketing of these devices if they do not have a different inlet and outlet coding system [23].

These various and severe incidents underline the importance of BVM system routine check and especially unidirectional valve reassembly after sterilization and cleaning by adequately trained personnel [21, 22, 24]. Single use built-in device may also be an alternative to avoid these disassembly problems.

### 5.4. Bag-Valve-Mask Ventilation Difficulty

Besides these technical incidents, BVM ventilation is quite not easy to perform in order to deliver adequate insufflations. Healthcare providers have no information on insufflated tidal volumes, ventilatory rates, gastric insufflation volumes, airway pressures, and leaks. These parameters are very important to appreciate helping the rescuer to adequately ventilate the patient. However, many studies have demonstrated that healthcare professionals trained in airway management provide to cardiac and/or respiratory arrest patient high ventilatory rates and inadequate ventilation volumes [25–27]. A study by Aufderheide et al. showed that experienced emergency medical personnel hyperventilated all patients with 37 ± 4 breaths/min (twice the recommendations) and none of them survived [25]. Furthermore, a recent bench study showed that hyperventilation occurred in simulated pediatric resuscitation with 40.6 ± 11.8 breaths/min compared to the recommended rate from 8 to 20 breaths/min by the Pediatric Advanced Life Support guidelines [28]. Recently, our research group has shown similar results in a bench study with a large and varied sample [29]. Another problem is the rapidly refilling bag and the emergency stressful situation which can induce a reflex in which rescuers tend to squeeze and deliver breath as soon as the bag reinflates [5]. These difficulties to perform adequate ventilation may lead to excessive insufflated volume and pressure. The latter induces high intrathoracic and airway pressures, which impair hemodynamics [30]. Furthermore, excessive ventilation favors gastric insufflation and subsequently pulmonary aspiration [31]. All these adverse effects may impact patients' survival.

These reports have pointed out the negative outcomes of human errors, which are usually the result of lack of experience and/or infrequent training. This leads to inadequate and inefficient ventilation according to the International Liaison Committee on Resuscitation (ILCOR) guidelines.

## 6. Conclusion

This literature review was focused on manual ventilation describing its history and the main devices currently used with their own advantages and hazards. Mouth-to-mouth resuscitation was described as early as the fifteen's century and progressively new ventilation techniques were developed leading to the concept of bag-valve-mask in the year 1950. Since that time, many hazards due to faulty valve or misassembly were reported in the literature as well as some difficulties to ensure efficient insufflation according to usual respiratory parameters. These malfunctions and difficulties lead to inadequate tidal volumes, induce high ventilation rates, and sometimes cause gastric insufflation. They also generate high airways and intrathoracic pressures. All these issues have a critical impact on patient survival. Trained healthcare workers should be in charge of BVM ventilation and the use of built-in devices will prevent disassembly problems and are safer than the reusable ones. Technological improvements are mandatory to increase the reliability, feasibility, and safety of bag-valve-mask ventilation. Throughout the past years, mechanical ventilation was improved drastically with a brand-new generation of ventilators that was developed, but little improvements were done for manual ventilation. Though the design and the engineering of Ambu valves have evolved, no major changes were done to Laerdal valves. The challenge is to develop devices and technologies that improve and secure the quality of manual ventilation.

## Figures and Tables

**Table 1:** Technical characteristics of Ambu and Laerdal valves.

	Ambu	Laerdal
Use		
Disassembly	Yes	Yes
Sterilizable	Yes	Yes
Single use	Yes	Yes
Mechanism		
Orthostatic	No	No
Manual occlusion	No	No
Rebreathing principle	No	No
Insufflation resistance	Yes	No
Spring mechanism	No	No
Operate to gas pressure	Yes	Yes
Monitoring		
Pressure relief valve	No (on the bag)	No (on the bag)
Direct communication risk (incoming gas/lung)	Yes	Yes
PEEP valve	Yes (adaptable)	Yes (adaptable)
Spirometry	No	Yes (adaptable)
Type		
Adult	Yes	Yes
Pediatric	Yes	Yes
